# Diagnostic utility of the urine neutrophil CD64 ratio for patients with urinary tract infections

**DOI:** 10.3389/fmed.2026.1769034

**Published:** 2026-04-09

**Authors:** Juan Jin, Chunyan Yao, Zhaosuo Hu, Runlin Yuan, Zhengxu Chen

**Affiliations:** 1 Department of Clinical Laboratory, The Second People’s Hospital of Hefei, Hefei, China; 2 Department of Clinical Laboratory, Hefei Hospital Affiliated to Anhui Medical University, Hefei, China

**Keywords:** blood, CD64, neutrophil, urinary tract infection, urine

## Abstract

**Objective:**

This study aimed to evaluate the clinical significance of the urine neutrophil CD64 ratio for diagnosing urinary tract infections (UTIs).

**Methods:**

The study enrolled 106 patients suspected to have UTIs and 30 healthy controls. Based on urine culture results and clinical symptoms, 58 confirmed UTI patients with positive urine culture results and clinical symptoms were included in the urine culture positive (UCP) group, while 48 suspected UTI patients with negative urine culture results but positive clinical symptoms were included in the urine culture negative (UCN) group. Quantitative urine bacterial culture was used to identify pathogenic species and bacterial load. Urine leukocyte esterase (U-LE), urine nitrite (U-NIT), and urine leukocyte count (U-LC) were detected by an automatic urine analyzer. Neutrophil CD64 median fluorescence intensity (MFI) in urine and blood was measured by flow cytometry, and the ratio of neutrophil CD64 MFI in urine (U-nCD64) and blood (B-nCD64) was calculated.

**Results:**

Among the 58 urine bacterial isolates, Gram-negative bacteria were the predominant species (74.14%), with *Escherichia coli* being the most common isolate (76.74%). Gram-positive bacteria (*Enterococcus* spp.) accounted for 25.86% of the total isolates. The U-nCD64 ratio was significantly higher in the UCP group than in the UCN group (*p* < 0.001). The UTI patient group had a significantly higher U-LC value than the healthy control (HC) group (*p* < 0.001, *p* < 0.001). The UCP and UCN groups had higher U-LE and U-NIT levels than the HC group. The U-nCD64 ratio was significantly positively correlated with U-LC (*p* = 0.0273) and U-LE (*p* = 0.0007). The area under the curve (AUC) value of the U-nCD64 ratio was 0.905 for diagnosing UTIs with the cutoff value, sensitivity, specificity, positive predictive value (PPV) and negative predictive value (NPV) of 0.89, 81.03, 89.58, 90.38 and 79.63%, respectively.

**Conclusion:**

The U-nCD64 ratio has the higher auxiliary value for diagnosing UTIs and is more conducive for the screening and early diagnosis of UTIs. Thus, the U-nCD64 ratio might be a promising biomarker for auxiliary diagnosis of UTIs.

## 1 Introduction

Urinary tract infection (UTI) is a common clinical infectious disease, reportedly affecting more than 404 million people globally in 2019 and occurring predominantly in individuals aged 30 to 34 years (1). UTIs are caused by microbial pathogens (mostly bacteria, with a small number of fungi) that multiply in the urethra and urine and invade the mucous membranes or tissues of the urinary system (2). Women and patients with indwelling catheters are highly susceptible to develop UTIs. Currently, urine bacterial culture is the standard diagnostic modality for UTIs; however, because this method is time-consuming, empirical treatment or non-standardized antimicrobial drug treatment is often used for some patients, which may promote the emergence of drug-resistant bacteria (3, 4). For asymptomatic patients, unconscious critically ill patients, infants and children without cognitive ability, UTI diagnosis mostly relies on the detection of routine urine infection indicators such as urine leukocyte count (U-LC), urine leukocyte esterase (U-LE), and urine nitrite (U-NIT); however, these indicators have poor diagnostic sensitivity and specificity, leading to inappropriate or delayed treatment and causing the patient’s condition to rapidly progress to chronic UTI or recurrent UTI (5). For example, in our study the PPV, NPV of U-LC, U-LE and U-NIT were 62.50 and 61.76%, 70.77 and 61.29%, 74.10 and 51.90%, respectively. Given this background, it is critical to develop new infection indices to guide clinical practice.

CD64 is a high-affinity immunoglobulin *γ* receptor I, mainly expressed on the surface of macrophages, monocytes, and dendritic cells and scarcely expressed on the surface of neutrophils; however, during active infection, CD64 expression on neutrophils is rapidly upregulated (6). The increased expression of CD64 on blood neutrophils is a new indicator for diagnosing infectious diseases and has a promising clinical application (7–9). Hence, in the present study, we evaluated the clinical value of urinary neutrophil CD64 for diagnosing UTIs.

## 2 Materials and methods

### 2.1 Patient selection

A total of 106 patients suspected to have UTIs and 30 healthy controls were included in this study. The inclusion criteria for patients were as follow: (1) showing symptoms of UTIs: pain on micturition, urinary urgency and frequency, and lower abdominal discomfort with or without systemic symptoms such as fever, renal area pain, nausea and vomiting. (2) abnormal or normal urinary bacterial cultures, (3) availability of urine routine examination results, and (4) does not receive antibiotic treatment in the past 48 h. Of the 106 patients, 58 confirmed UTI patients with positive urine culture results and bacterial colony count exceeding 10^5^/mL urine were included in the urine culture positive (UCP) group, and 48 suspected UTI patients with negative urine culture results but showing UTI symptoms were included in the urine culture negative (UCN) group. The following patients were excluded from the study: (1) those with autoimmune disease, HIV, cancer, or end-stage disease, (2) those with indwelling catheter or removal of the catheter or receive antibiotic treatment within the past 48 h, and (3) those who refused to give informed consent to participate in the study. Thirty individuals who visited the hospital for health checkups were selected as the healthy control group; these individuals had normal physical examination results and negative urine culture results and had no symptoms of UTIs or other severe diseases. This study was approved by the Research Ethics Committee of the Second People’s Hospital of Hefei City (Approval No. 2023–092) and all participants signed the informed consent form.

### 2.2 Sample collection and UTI detection

Two random mid-stream urine samples were collected from the study subjects. One sample was collected in a 10 mL plastic tube for measuring urine routine indicators by using an automated urine analyzer (FUS-2000, China) and for detecting urine neutrophil CD64 level by flow cytometry with a flow cytometer (FACSCalibur, Becton Dickinson, USA). The other sample was collected in a sterile tube for bacterial culture and identification. The samples were processed in the laboratory with 2 h of collection or stored at 4 °C until delivery and processed no more than 6 h after initial collection. For bacterial culture, the urine specimens were inoculated on agar plates, and the plates were incubated overnight at 37 °C in a 5% CO_2_ incubator. The isolated bacterial strain was identified using an automated rapid bacterial identification system (VITEK^®^ 2 COMPACT, Biomerieus, France) and a mass spectrometry detection system (IVD MALDI Biotyper^®^, Bruker, Germany). Additionally, 2 mL of venous blood was collected in EDTA-coated anticoagulation tubes, mixed, and subjected to blood neutrophil CD64 estimation within 6 h.

### 2.3 Neutrophil CD64 detection by flow cytometry

Two monoclonal antibodies conjugated to Peridinin Chlorophyll Protein (PerCP) and Phycoerythrin (PE) was used (CD45-PerCP and CD64-PE). Based on the result of U-LC and the volume of urine specimen collected, the concentration of urine leukocytes was adjusted to 1 × 10^6^ cells/mL. Next, 50 μL of the urine specimen was incubated in the dark for 20 min at room temperature with CD45-PerCP and CD64-PE (test tube) or CD45-PerCP and isotype control of CD64-PE (isotype control tube). The cells were then washed with 2 mL of phosphate-buffered saline (PBS). Finally, 300 μL of PBS was added to each tube to resuspend the cells, and urine neutrophil CD64 median fluorescence intensity (MFI) was determined using a flow cytometer (FACSCalibur, Becton Dickinson, USA). Before test, flow cytometer setup and calibration were based on the manufacturer’s instructions. For testing the blood sample, 50 μL of anticoagulated whole blood specimen was incubated with CD45-PerCP and CD64-PE (test tube) or CD45-PerCP and isotype control of CD64-PE (isotype control tube). Red blood cells were then lysed with BD FACS™ Lysing Solution (BD Biosciences, San Jose, CA, USA). The subsequent processing steps were the same as those for the urine specimen. Fluorescent antibodies for flow cytometry (anti-human CD45-PerCP, anti-human CD64-PE, and isotype control) were purchased from Beijing Tongsheng Times Biotechnology Corporation.

Flow cytometry data were acquired using CellQuest software and analyzed with FlowJo 10.0 software. First, neutrophils were gated in the scatter plot with CD45-PerCP on the abscissa and side scatter on the ordinate. Subsequently, in the histogram of CD64-PE, the MFI of PE was adjusted to within 10 by using the isotype control tube. Next, neutrophil CD64 MFI in the test tube was detected and analyzed. Data for up to 10,000 events were obtained in each tube. The neutrophil CD64 ratio was calculated as follows: (CD64 MFI_test_–CD64 MFI_control_)/CD64 MFI_control_.

### 2.4 Statistical analysis

GraphPad Prism 8.0 software was used for statistical analysis. The results were compared using ANOVA, Mann–Whitney, Kruskal-Wallis, and chi-square tests. Spearman’s rank correlation test was used for correlation analysis. Receiver operating characteristic (ROC) analysis and Youden J statistic were used to calculate the cutoff values, sensitivity, specificity, positive predictive value (PPV), and negative predictive value (NPV). A *p*-value of <0.05 was considered statistically significant.

## 3 Results

### 3.1 Distribution of bacterial species isolated from urine samples

Table 1 shows the distribution of Gram-negative and Gram-positive bacteria isolated from the urine samples of UCP group patients. Among the 58 urine bacterial isolates, Gram-negative bacteria were the predominant species (74.14%), Gram-positive bacteria (*Enterococcus* spp.) accounted for 25.86% of the total isolates. Among the Gram-negative isolates, *Escherichia coli* (76.74%) was identified as the leading organism, the second most frequent bacterial species were *Acinetobacter* spp. and *Proteus* spp. (6.98% each), followed by *Klebsiella* spp. and *Pseudomonas aeruginosa* (4.65% each) (Table 1).

**Table 1:** Distribution of gram-negative and gram-positive bacteria in urine samples from the UCP group.

Bacteria	Gram-negative (*n* = 43)	Gram-positive (*n* = 15)	Total (*n* = 58)
*Escherichia coli*	33 (76.74%)	/	33 (56.90%)
*Acinetobacter* spp.	3 (6.98%)	/	3 (5.17%)
*Proteus* spp.	3 (6.98%)	/	3 (5.17%)
*Klebsiella* spp.	2 (4.65%)	/	2 (3.45%)
*Pseudomonas aeruginosa*	2 (4.65%)	/	2 (3.45%)
*Enterococcus* spp.	/	15 (100%)	15 (25.86%)

### 3.2 Patient characteristics

The median values and interquartile ranges (IQRs) of participants’ age was 52.50 (44.75–58.00) years in the UCP group, 57.00 (52.50–66.75) years in the UCN group, and 51.50 (42.00–62.00) years in the HC group. The male-to-female ratios were 0.71:1, 0.60:1, and 0.67:1 in the UCP, UCN, and HC groups, respectively. The UCP, UCN, and HC groups showed similar age and gender ratios, with no significant differences among the three groups (Table 2).

**Table 2:** Comparison of patient characteristics of the UCP, UCN, and HC groups.

Parameters	UCP group, *n* = 58	UCN group, *n* = 48	Healthy control, *n* = 30
Gender (Male/Female)	0.71:1 (24/34)	0.60:1 (18/30)	0.67:1 (12/18)
Age, median (IQRs)	52.50 (44.75–58.00)	57.00 (52.50–66.75)	51.50 (42.00–62.00)
U-nCD64 ratio median (IQRs)	1.60^a^ (1.04–3.00)	0.36 (0.20–0.72)	ND
B-nCD64 ratio median (IQRs)	7.84 (6.25–9.68)	6.40 (4.80–8.92)	5.74 (4.46–8.39)
U-LC (μL) median (IQRs)	424.5^b^ (247.0–807.8)	302.0^c^ (197.3–595.3)	0.0 (0.0–0.0)
U-LE, median (IQRs)	3+^a,b^ (3 + −3+)	2+^c^ (1 + −3+)	0 (0–0)
U-NIT (positive %)	34.48%^a,b^	14.58%^c^	0.00%

UCP, urine culture positive; UCN, urine culture negative; IQRs, interquartile ranges; U-nCD64,urine neutrophil CD64; B-nCD64, blood neutrophil CD64; U-LC, urine leukocyte count; U-LE, urine leukocyte esterase; U-NIT, urine nitrite; ND, not detected.

^a^U-nCD64 ratio, U-LE, and U-NIT were significantly higher in the UCP group than in the UCN group (*p* < 0.001, *p* = 0.005, and *p* = 0.025, respectively).

^b^U-LC, U-LE, and U-NIT were significantly higher in the UCP group than in the HC group (*p* < 0.001, *p* < 0.001, and *p* < 0.001, respectively).

^c^U-LC, U-LE, and U-NIT were significantly higher in the UCN group than in the HC group (*p* < 0.001, *p* < 0.001, and *p* = 0.039, respectively).

### 3.3 Neutrophil CD64 expression was upregulated in urine but not in blood in the UCP group

**Figure 1:**
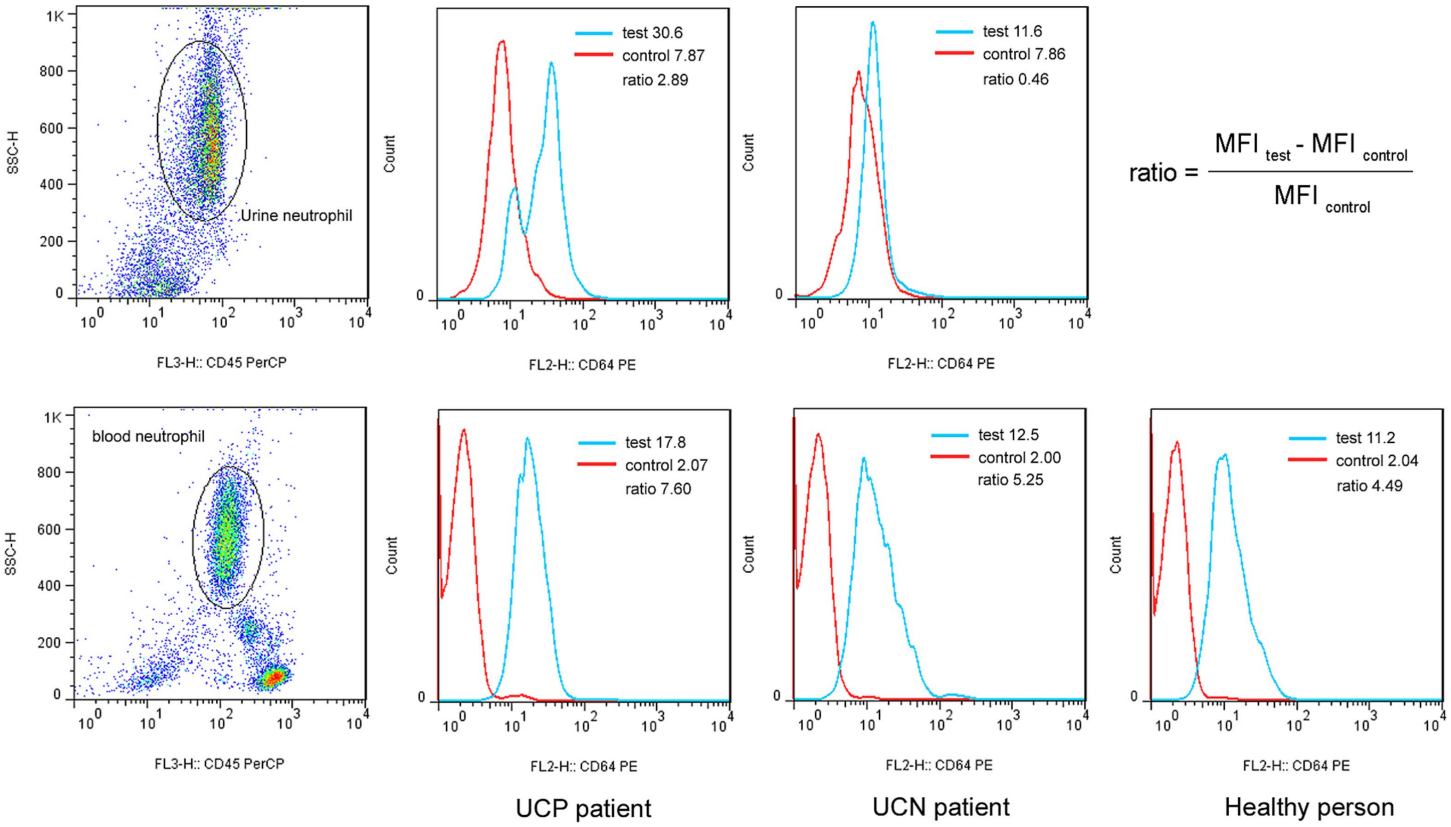
Histograms showing the neutrophil CD64-PE expression levels in urine (top) and blood (bottom) from a UCP patient, a UCN patient, and a healthy control. MFI, median fluorescence intensity; UCP, urine culture positive; UCN, urine culture negative.

**Figure 2:**
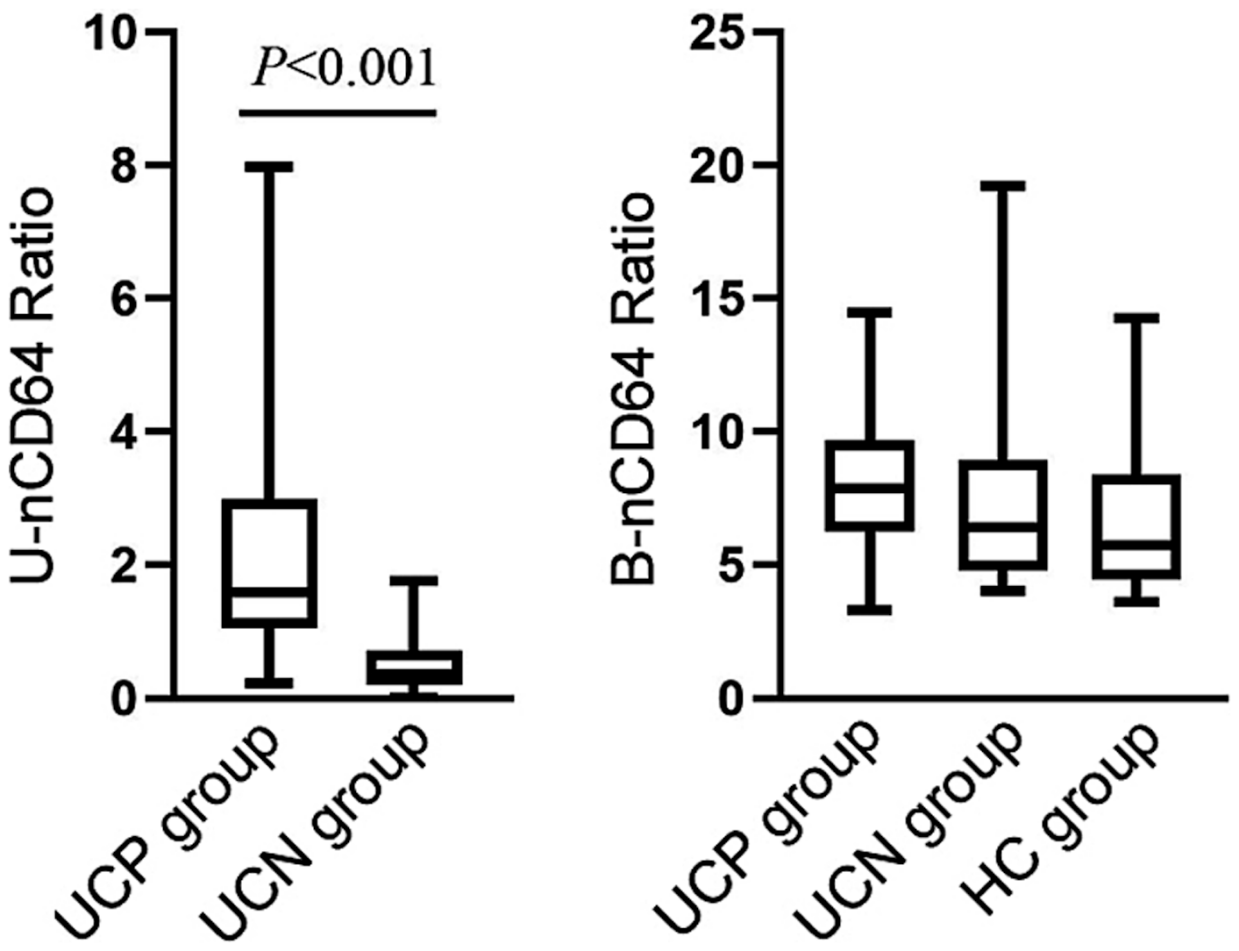
Comparison of the U-nCD64 and B-nCD64 ratios among the UCP, UCN, and HC groups. UCP group patients showed a higher U-nCD64 ratio than UCN group patients. U-nCD64, urine neutrophil CD64; B-nCD64, blood neutrophil CD64; UCP, urine culture positive; UCN, urine culture negative; HC, healthy control.

The UCP group showed a significantly higher U-nCD64 ratio than the UCN group (*p <* 0.001); however, no significant difference in the B-nCD64 ratio was observed among the UCP, UCN, and HC groups (Figures 1, 2). Additionally, UCP and UCN group participants had a higher U-LC count and higher U-LE and U-NIT levels compared to HC group participants. The test findings are shown in Table 2.

### 3.4 Correlation analysis

**Figure 3:**
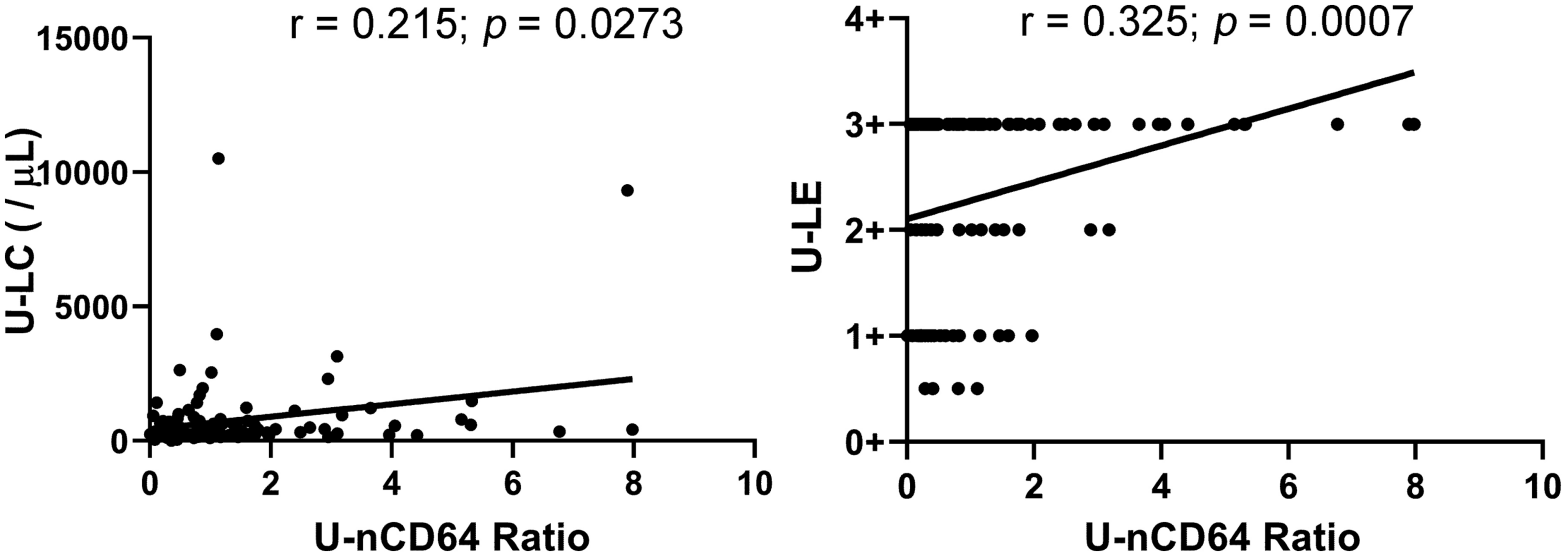
Correlation between the U-nCD64 ratio and U-LC, U-LE. U-nCD64, urine neutrophil CD64, U-LC, urine leukocyte count; U-LE, urine leukocyte esterase.

Spearman’s correlation analysis was performed for all possible combinations of the U-nCD64 ratio and U-LC, U-LE, and U-NIT in the UCP and UCN groups. Though U-nCD64 ratio showed a significant positive correlation with U-LC and U-LE (*r* = 0.215, *p* = 0.0273; *r* = 0.325, *p* = 0.0007, respectively) (Figure 3), but the correlation was weak, as the correlation coefficients were below 0.4. No significant correlation was detected between U-nCD64 ratio and U-NIT (*r* = 0.083, *p* = 0.397).

### 3.5 ROC curve analysis

**Figure 4:**
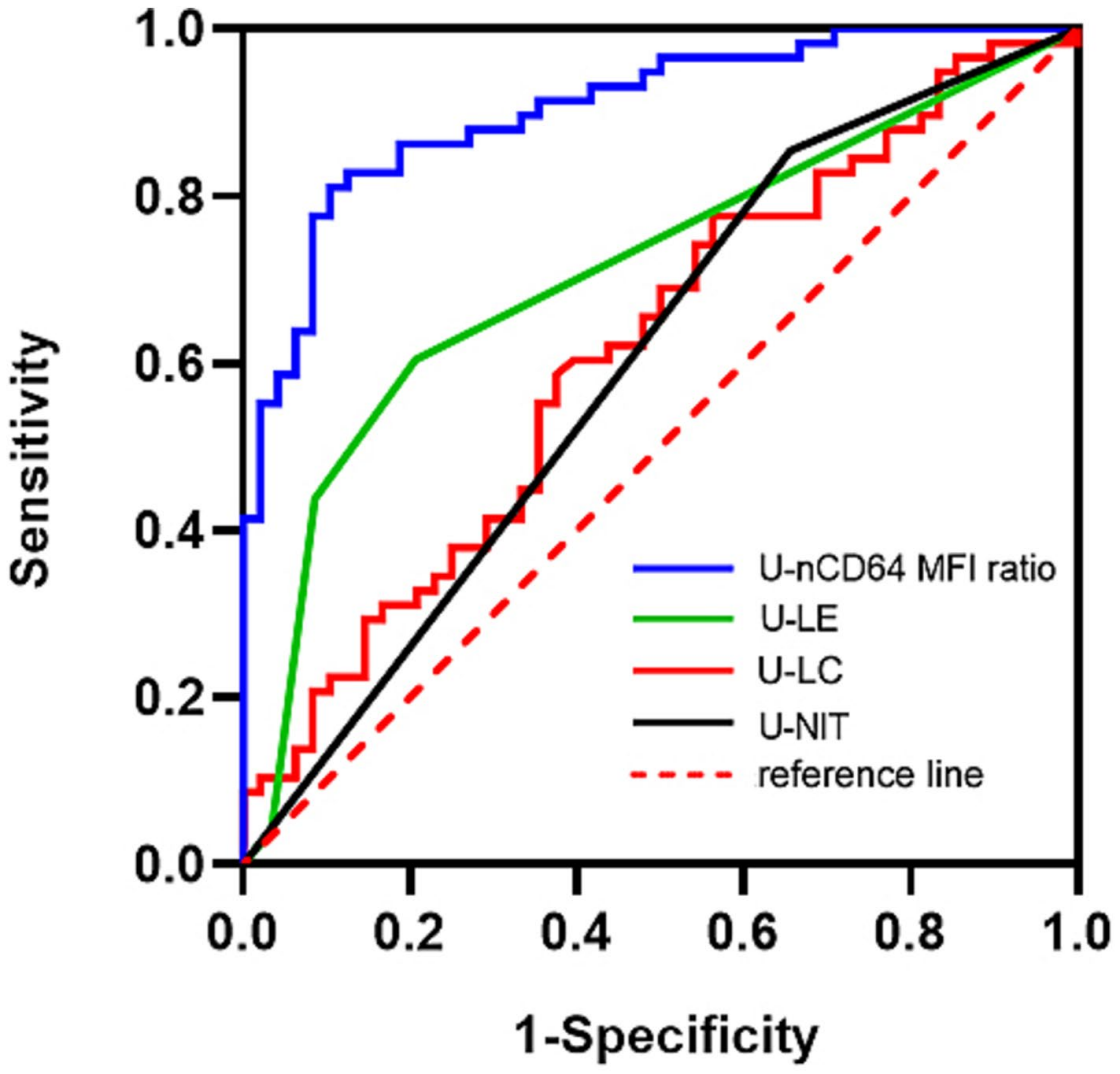
AUC values based on the ROC curve analysis of the U-nCD64 ratio, U-LE, U-LC, and U-NIT for UTIs. U-nCD64, Urine neutrophil CD64; U-LC, urine leukocyte count; U-LE, urine leukocyte esterase; U-NIT, urine nitrite; MFI, median fluorescence intensity.

The ROC curve analysis yielded the following results for the U-nCD64 ratio, U-LE, U-LC and U-NIT: (1) U-nCD64 ratio: optimum diagnostic cutoff value, 0.89; AUC, 0.905 (95% CI: 0.849–0.960); sensitivity, 81.03% (95% CI: 69.15–89.07%); specificity, 89.58% (95% CI: 77.83–95.47%), PPV, 90.38%; NPV, 79.63%, (2) U-LE: optimum diagnostic cutoff value, 2.5+; AUC, 0.712 (95% CI: 0.610–0.814); sensitivity, 60.42% (95% CI: 46.31–72.98%); specificity, 79.31% (95% CI: 67.23–87.75%); PPV, 70.77%; NPV, 61.29%, (3) U-LC: optimum diagnostic cutoff value, 242/μL; AUC, 0.615 (95% CI: 0.508–0.723); sensitivity, 77.59% (95% CI: 65.34–86.41%); specificity, 43.75% (95% CI: 30.70–57.72%); PPV, 62.50%; NPV, 61.76%, and (4) U-NIT: optimum diagnostic cutoff value, 0.5; AUC, 0.600 (95% CI: 0.492–0.707); sensitivity, 85.42% (95% CI: 72.83–92.75%); specificity, 34.48% (95% CI: 23.56–47.33%); PPV, 74.10%; NPV, 51.90%. The U-nCD64 ratio exhibited the highest sensitivity and specificity in the auxiliary diagnosis of UTIs (Table 3 and Figure 4).

**Table 3:** ROC curve analysis of the U-nCD64 ratio, U-LC, U-LE, and U-NIT.

Index	AUC (95% CI)	*p* value	Cutoff value	Sensitivity (%, 95 CI)	Specificity (%, 95 CI)	YI	PPV (%)	NPV (%)
U-nCD64 ratio	0.905 (0.849–0.960)	<0.0001	0.89	81.03 (69.15–89.07)	89.58(77.83–95.47)	0.71	90.38	79.63
U-LE	0.712 (0.610–0.814)	0.0002	2.5+	60.42 (46.31–72.98)	79.31 (67.23–87.75)	0.40	70.77	61.29
U-LC (μL)	0.615 (0.508–0.723)	0.0419	242	77.59 (65.34–86.41)	43.75 (30.70–57.72)	0.21	62.50	61.76
U-NIT	0.600 (0.492–0.707)	0.0787	0.5	85.42 (72.83–92.75)	34.48 (23.56–47.33)	0.20	74.10	51.90

ROC, receiver operating characteristic; AUC, area under the curve; U-nCD64, urine neutrophil CD64; U-LC, urine leukocyte count; U-LE, urine leukocyte esterase; U-NIT, urine nitrite; YI, Youden index; PPV, positive predictive value; NPV, negative predictive value.

## 4 Discussion

UTIs are caused by the invasion of the urinary system by various pathogens and are a relatively common infection in clinical settings. Although urine bacterial culture is recognized as the standard diagnostic modality for UTIs, the delay in obtaining the results does not support its application for disease screening and early diagnosis. While routine urine infection indicators such as U-LC, U-LE and U-NIT are more widely used, they have poor sensitivity and specificity, making their clinical diagnostic value limited. In recent years, urine heparin-binding protein (HBP) has received increasing attention as a new indicator to diagnose UTIs (10–13). Taha et al. reported that in UTI, urine-HBP showed a high diagnostic value in differentiating bacterial UTI infection from nonbacterial UTI infection at the cutoff value of 32.868 ng/mL with sensitivity and specificity of 87% each (10). Other humoral biomarkers, such as interleukin (IL)-6, IL-8, and secreted tumor necrosis factor are also potentially useful to diagnose UTIs (14, 15). However, to date, few studies have reported new indicators to diagnose UTIs at the cellular level.

The B-nCD64 has a higher diagnostic value than procalcitonin and IL-6 for diagnosing sepsis (16). Ascitic fluid neutrophil CD64 is beneficial for the rapid diagnosis of bacterial infection in patients with ascites, enabling prompt administration of antibiotic treatment (7). Additionally, cerebrospinal fluid neutrophil CD64 has a high diagnostic value for diagnosing bacterial ventriculitis in children (17). However, the application of U-nCD64 for the diagnosis of bacterial UTIs has not yet been reported and deserves in-depth investigation. Hence, in the present study, we comprehensively evaluated the clinical application value of urinary neutrophil CD64 expression level, i.e., U-nCD64 ratio, in the auxiliary diagnosis of UTIs for the first time.

One of the critical findings of the present study is that the U-nCD64 ratio was significantly higher in the UCP group than in the UCN group. Neutrophils play a crucial role in the antimicrobial defense and mitigation of UTIs. Following their activation at the site of infection, these innate immune cells phagocytose and neutralize an invading pathogen. Neutrophils also release bioactive compounds such as antimicrobial peptides and proteins stored in neutrophil granules and reactive oxygen species. Our study further confirmed that urinary neutrophils are in a highly activated state in patients with UTIs.

To clarify whether the UCP and UCN groups exhibited differences in the B-nCD64 ratio identical to that in the U-nCD64 ratio, we also measured the B-nCD64 ratio. The UCP and UCN groups showed no significant difference in the B-nCD64 ratio; moreover, a significant difference in the B-nCD64 ratio was also not observed between the patient and HC groups. These results suggest that the activated state of neutrophils is found only in urine but not in blood of patients with UTIs. UTIs are generally classified as either uncomplicated UTIs (uUTIs) or complicated UTIs (cUTIs) (18). The symptomatology of uUTIs and cUTIs varies according to the location of these infections; common symptoms of uUTIs are urinary frequency, urinary urgency, urinary pain, etc., while cUTIs more commonly manifest as pain in the renal area and fever (18, 19). Based on these reports, we hypothesized that the blood neutrophils of patients with cUTIs might be in an activated state, which requires further investigations. Moreover, we reviewed the medical records of the UCP group patients, only five patients have fever, none have renal area pain, nausea and vomiting, so the majority of UCP group patients were uUTIs in our study.

U-LC, U-LE, and U-NIT are widely used in clinical practice; however, their levels could be influenced by a variety of conditions, leading to poor specificity for diagnosing UTIs. The sensitivity of these indicators especially U-NIT is not promising too. U-NIT could detect nitrite reductase-containing bacteria, but some common bacteria in UTIs cannot reduce nitrate to nitrite, the number of false-negative cases is significant. Additionally, the cause of the false-negative results also include a lack of a source of nitrate in the diet, the time that urine is stored in the bladder in contact with bacteria and the presence of external interfering chemical, such sa ascorbic acid. In the present study we compared and analyzed the correlations between the U-nCD64 ratio and U-LC, U-LE, and U-NIT. The results showed that the U-nCD64 ratio was significantly but weak positively correlated with U-LC and U-LE, but not with U-NIT. We hypothesized that the U-nCD64 ratio, U-LC, and U-LE are related to the presence of neutrophils in urine. The U-nCD64 ratio reflects the activation status of neutrophils, U-LC denotes the number of neutrophils, and U-LE is a specific enzyme contained in neutrophils; thus, a correlation exists between them.

Further evaluation of the diagnostic efficacy of the U-nCD64 ratio, U-LC, U-LE, and U-NIT revealed that the AUC value (0.905) of the U-nCD64 ratio was the highest for diagnosing UTIs, with the cutoff value, sensitivity, specificity, PPV and NPV of 0.89, 81.03, 89.58, 90.38 and 79.63%, respectively. This finding suggests that the U-nCD64 ratio has a higher diagnostic efficacy than the other three indices, making its clinical application more valuable. It must be mentioned that in our study UCN group has heterogeneity, which might reduced the sensitivity of the U-nCD64 ratio. UTIs are inflammatory reactions of the urinary tract mainly caused by multispecies microorganisms– including bacteria, fungi, and viruses, so non-bacterial UTIs patients which were included in the UCN group in our study. Some UTI patients who might have self-treated with antibiotics also might be included in the UCN group. In addition, improper collection or handling of the specimen leading to false negative results, are included in the UCN group too.

The present study established that the U-nCD64 ratio is a clinically significant indicator for the auxiliary diagnosis of UTIs. First, our study showed that the U-nCD64 ratio has the highest sensitivity and specificity among the routine urinary markers for diagnosing bacterial UTIs. Second, the U-nCD64 ratio testing period is approximately 2 h, making this test more feasible for application in bacterial UTI screening and early diagnosis compared to urine bacterial culture. Finally, the U-nCD64 ratio could facilitate early administration of empirical antimicrobial drug treatment for bacterial UTIs. Together with the promotion and popularization of flow cytometry in clinical application, the U-nCD64 ratio could serve as an optimal indicator for the clinical auxiliary diagnosis of UTIs.

There are some limitations of the present study. It is a single-center study, the sample size of each groups is small. Subgroup analysis was not performed for UTI patients. Moreover, the UCN group may have misclassification bias, because patients with positive symptoms but negative urine cultures, which could indicate non-bacterial UTIs, previous antibiotic exposure, or culture-negative infections, are included in the UCN group. This bias weak the sensitivity of this method to a certain extent. In addition, this study does not assess the comorbidities such as diabetes or renal illness, which might confounder the results. Absence of a cohort for external validation is another limitation of this study. Finally, flow cytometry is not popularization in clinical application, which restricted use of this study.

In conclusion, the present study demonstrates that the U-nCD64 ratio has a higher clinical value for the auxiliary diagnosis of UTIs compared to U-LE, U-NIT, and U-LC and has more feasibility for application in the screening and early diagnosis of UTIs compared to urine bacterial culture. Thus, the U-nCD64 ratio could be a promising biomarker for the auxiliary diagnosis of UTIs.

## Data Availability

The original contributions presented in the study are included in the article/supplementary material, further inquiries can be directed to the corresponding author/s.

## Glossary

UTIs: Urinary tract infections
UCP: Urine culture positive
UCN: Urine culture negative
U-LE: Urine leukocyte esterase
U-NIT: Urine nitrite
U-LC: Urine leukocyte count
U-nCD64: Urine neutrophil CD64
B-nCD64: Blood neutrophil CD64
AUC: Area under the curve
ROC: Receiver operating characteristic
PBS: Phosphate-buffered saline
MFI: Median fluorescence intensity
IQR: Interquartile range
HC: Healthy control
HBP: Heparin-binding protein
uUTIs: Uncomplicated UTIs
cUTIs: Complicated UTIs
YI: Youden index
PPV: Positive predictive value
NPV: Negative predictive value
PerCP: Peridinin chlorophyll protein
PE: Phycoerythrin
